# Ibaraki’s Amabie-chan usage and its association with infection prevention behavior and fear of COVID-19: a cross-sectional preliminary survey of the Tsukuba Salutogenic Occupational Cohort Study

**DOI:** 10.1265/ehpm.22-00052

**Published:** 2022-04-16

**Authors:** Daisuke Hori, Yuichi Oi, Shotaro Doki, Tsukasa Takahashi, Tomohiko Ikeda, Yu Ikeda, Yo Arai, Kei Muroi, Hiroaki Sasaki, Mami Ishitsuka, Asako Matsuura, Wyi Go, Ichiyo Matsuzaki, Shinichiro Sasahara

**Affiliations:** 1 Occupational and Aerospace Psychiatry Group, Faculty of Medicine, University of Tsukuba, 1-1-1 Tennodai, Tsukuba, Ibaraki 305-8575, Japan; 2 Occupational Health Committee, Tsukuba Science City Network, 2-20-5 Takezono, Tsukuba, Ibaraki 305-0032, Japan; 3 Graduate School of Comprehensive Human Sciences, University of Tsukuba, 1-1-1 Tennodai, Tsukuba, Ibaraki 305-8575, Japan; 4 International Institute for Integrative Sleep Medicine, University of Tsukuba, 1-1-1 Tennodai, Tsukuba, Ibaraki 305-8575, Japan

**Keywords:** Ibaraki’s Amabie-chan, Infection control, Mobile applications, SARS-CoV-2

## Abstract

**Background:**

Ibaraki’s Amabie-chan is a COVID-19 infection control system unique to Ibaraki prefecture, Japan. It requires residents to register each time they visit events, commercial facilities, and restaurants. The number of registrations has been limited, and its function alerting about people positive for COVID-19 infection seems not to be working. Nevertheless, registration with the system might have some impact on the user’s behavior. In the current preliminary survey, the possible impact of Ibaraki’s Amabie-chan on infection prevention behavior and fear of COVID-19 was investigated.

**Methods:**

A cross-sectional, web-based, anonymous, and self-administered survey was conducted at two workplaces in Tsukuba Science City, Ibaraki, Japan. The first survey was conducted at one of the workplaces in November 2020, and the second survey, at the other workplace in February 2021. Variables of interest were sex, age group, marital status, employment status, Ibaraki’s Amabie-chan use, COVID-19 Contact-Confirming Application use, ten items of infection prevention behaviors, and fear of COVID-19. Hierarchical linear regression analysis was performed.

**Results:**

In both surveys, use of Ibaraki’s Amabie-chan was significantly associated with COCOA use and with “physical condition management such as body temperature measurement.” No association was found with other infection prevention behaviors or with fear of COVID-19.

**Conclusions:**

Our findings did not provide sufficient evidence for the effectiveness of Ibaraki’s Amabie-chan in regard to users’ infection control behavior. Further detailed study is needed to investigate the effectiveness in terms of infection prevention and the cost-effectiveness of Ibaraki’s Amabie-chan.

## Background

During the COVID-19 pandemic, it has become a social phenomenon in Japan to share illustrations of Amabie, Japanese *yōkai*, in the hope that COVID-19 will go away [1]. Ibaraki’s Amabie-chan [2], named after Amabie, is a system unique to Ibaraki prefecture that has the aim of “preventing the spread of the COVID-19 virus by not only supporting business owners who follow the prefecture’s guidelines and work on infection preventive measures, but also by notifying those who may have come in contact with an infected person in case a confirmed case has been discovered.” It was launched in June 2020, and the app version has been available since February 2021.

Ibaraki’s Amabie-chan is similar to COVID-19 Contact-Confirming Application (COCOA) which is operated nationwide. However, they differ in several respects. First, Ibaraki’s Amabie-chan does not use the Exposure Notification API built by Google and Apple. Second, event organizers or business owners have to apply to Ibaraki prefectural offices to obtain a QR code specifically linked to their events or shops. They have to post at their venues a pledge alongside the QR code stating that they are taking thorough measures to prevent infection. Third, visitors to an event or shop have to register through the QR code using their smartphones each time they visit. In this way, the places and the dates of their visits are recorded. After that, the prefecture office will send an alert to notify the visitors if a COVID-19-positive person has been found to have used the facility on the same day.

For an alert system like COCOA to work, a fairly high percentage of people need to actively use it. COCOA did not work as expected due to a limited number of active users [3, 4]. The same is true for Ibaraki’s Amabie-chan. Nevertheless, the registration might have had some effect on users’ perceptions and behaviors. For example, the use of Ibaraki’s Amabie-chan may serve as a reminder to users to take preventive action against infection. Furthermore, looking at the pledge made by shops may link to a mitigation in fear of COVID-19. We conducted a survey to examine the association between use of Ibaraki’s Amabie-chan and implementation of infection prevention behaviors and mitigation of fear of COVID-19.

## Methods

A cross-sectional, web-based, anonymous, and self-administered survey was conducted at two workplaces in Tsukuba Science City, Ibaraki, Japan. The survey at workplace-A, a research institute for basic science, was conducted in November 2020, when the number of COVID-19 cases was low and stable. The survey at workplace-B, an office of the local government, was conducted in February 2021, when Ibaraki was under a prefecture-initiated declared state of emergency. During that COVID-19 wave, the number of daily-reported positive cases in Ibaraki prefecture peaked on January 15, at 159 cases.

An email containing a link to an online questionnaire was sent to about 2500 eligible workers in total. A total 456 respondents’ demographic information is described elsewhere [5]. In the current study, participants who had the choice to use Ibaraki’s Amabie-chan/COCOA were eligible for the analysis. Thus, the participants who did not know about them or who did not have a compatible device were excluded. Participants aged older than 70 years and those whose sex was unspecified were also excluded.

For use of Ibaraki’s Amabie-chan, we asked “Do you know about Ibaraki’s Amabie-chan?” In the case of “Yes” responses, respondents proceeded to the following question, “Choose which of the following best describes your use of Ibaraki’s Amabie-chan.” 3 = “I have registered multiple times,” 2 = “I have registered once,” 1 = “I have never registered although I use a compatible device,” and N/A = “I don’t use a compatible device.” Similarly, use of COCOA was dichotomized into 2 = “I have installed the app and I am using it” and 1 = other options except lack of a compatible device. Ten items of infection prevention behaviors were developed by the authors on the basis of the “New Normal Lifestyle” [6]. We asked “How often do you practice the following new ways of living? Looking back over the past 30 days, choose the one that best applies to you.” Each item was followed by five options and scored accordingly: from 5 = “Almost always,” to 1 = “Never.” The higher score indicates more frequent acts of infection prevention behaviors. The Fear of COVID-19 Scale, comprising seven items with a five-point Likert scale, was used [7]. A higher total score indicates a higher level of fear of COVID-19.

We showed the Spearman’s rank coefficient correlation results. We also performed a hierarchical linear regression analysis, with use of Ibaraki’s Amabie-chan as the dependent variable. In step 1, sex, age group, and marital status were forcedly entered. In step 2, a forward stepwise selection method was used (likelihood ratio) for COCOA use, 10 items of infection prevention behaviors, and total score of fear of COVID-19. All statistical tests were two-tailed, with probability values <0.05 considered significant. IBM SPSS for Windows (version 27.0; IBM Corporation, Armonk, NY, USA) was used.

## Results

In total, 159 responses (34.6% female) from workplace-A and 188 responses (39.4% female) from workplace-B were analyzed. The most frequent age group was 50–59 years for both workplaces. Respectively for workplace-A/B, 11.9/30.3% of the respondents had registered with Ibaraki’s Amabie-chan more the once, and 11.3/17.6% had done so only once. The difference between the two workplaces was probably due to the three-month interval in conducting the survey. Table 1 shows the correlation coefficients between pairs of variables of interest. Table 2 demonstrates the results of the hierarchical regression analysis. For both workplace-A and workplace-B respondents, Ibaraki’s Amabie-chan use was significantly associated with COCOA use and “Physical condition management such as body temperature measurement.” No other infection preventive behavior or fear of COVID-19 was selected into the regression equation.

**Table 1:** Distributions and correlation coefficients between Ibaraki’s Amabie-chan use and infection prevention behaviors.

	**Range**	**Median of worksite-A^a^**	**Median of worksite-B^b^**	**Spearman’s rank correlation coefficients^c^**												

				**1.**	**2.**	**3.**	**4.**	**5.**	**6.**	**7.**	**8.**	**9.**	**10.**	**11.**	**12.**	**13.**
1. Ibaraki’s Amabie-chan use	1–3	1	1	1	0.29^**^	0.05	0.05	0.09	0.17^*^	0.12	0.07	0.13	0.12	0.27^**^	0.20^**^	0.03
2. COCOA use	1–2	1	1	0.34^**^	1	0.14	0.01	−0.04	0.08	0.07	−0.03	0.02	0.03	0.11	0.17^*^	−0.02
3. Frequent hand washing and sanitizing	1–5	5	5	0.04	0.01	1	0.24^**^	0.28^**^	0.48^**^	0.43^**^	0.38^**^	0.26^**^	0.20^**^	0.27^**^	0.26^**^	0.06
4. Wearing a mask	1–5	5	5	−0.07	0.11	0.34^**^	1	0.43^**^	0.30^**^	0.22^**^	0.21^**^	0.20^**^	0.07	0.09	0.06	−0.04
5. Thorough cough etiquette	1–5	5	5	0.07	0.04	0.40^**^	0.44^**^	1	0.29^**^	0.28^**^	0.27^**^	0.26^**^	0.15^*^	0.18^*^	0.08	0.00
6. Keeping physical distance	1–5	4	4	−0.02	0.02	0.37^**^	0.21^**^	0.28^**^	1	0.72^**^	0.59^**^	0.61^**^	0.30^**^	0.30^**^	0.26^**^	0.06
7. Avoiding closed spaces	1–5	4	4	−0.03	−0.13	0.42^**^	0.16	0.39^**^	0.59^**^	1	0.60^**^	0.65^**^	0.27^**^	0.27^**^	0.26^**^	0.06
8. Avoiding crowded places	1–5	4	5	−0.02	−0.05	0.36^**^	0.23^**^	0.26^**^	0.65^**^	0.65^**^	1	0.65^**^	0.30^**^	0.27^**^	0.29^**^	−0.01
9. Avoiding close-contact settings	1–5	4	4	−0.01	0.00	0.36^**^	0.14	0.16^*^	0.68^**^	0.62^**^	0.69^**^	1	0.29^**^	0.23^**^	0.32^**^	0.03
10. Appropriate lifestyle habits such as exercise, diet, and smoking cessation according to one’s health condition	1–5	4	4	−0.03	−0.08	0.19^*^	0.07	0.18^*^	0.25^**^	0.26^**^	0.28^**^	0.23^**^	1	0.09	0.16^*^	−0.06
11. Physical condition management such as body temperature measurement	1–5	3	4	0.18^*^	0.07	0.16^*^	0.15	0.12	0.26^**^	0.13	0.23^**^	0.26^**^	0.15	1	0.41^**^	0.13
12. Keeping a record of the places you visited and people you met	1–5	2	2	0.16	0.05	0.14	−0.01	0.07	0.13	0.17^*^	0.14	0.14	0.13	0.26^**^	1	0.27^**^
13. Fear of COVID-19	7–35	16	18	0.01	0.02	0.15	0.18^*^	0.03	0.10	0.07	0.12	0.17^*^	−0.04	0.15	0.04	1

^a^Worksite-A: Survey was conducted in November 2020. Data from 159 respondents were analyzed.

^b^Worksite-B: Survey was conducted in February 2021. Data from 188 respondents were analyzed.

^c^The results of worksite-A are shown in the lower left, and those of worksite-B, in the upper right.

^*^P < 0.05; ^**^P < 0.01.

COCOA, COVID-19 Contact-Confirming Application.

**Table 2:** Results of hierarchical linear regression analysis, Ibaraki’s Amabie-chan use^a^ as dependent variable

		**Worksite-A^b^**			**Worksite-B^c^**		
	
		**B**	**95% CI**	***P*-value**	**B**	**95% CI**	***P*-value**
Model 1	Sex^d^	−0.17	−0.40, 0.06	0.15	−0.02	−0.29, 0.25	0.90
	Age group^e^	0.02	−0.07, 0.12	0.66	0.06	−0.07, 0.19	0.37
	Marital status^f^	−0.01	−0.27, 0.25	0.94	0.11	−0.23, 0.45	0.53
Model 2	2. COCOA use	0.45	0.24, 0.67	<0.001	0.60	0.31, 0.89	<0.001
Model 3	11. Physical condition management such as body temperature measurement	0.09	0.01, 0.18	0.030	0.15	0.05, 0.25	0.002

^a^Coded as 3 = “I have registered multiple times,” 2 = “I have registered once,” and 1 = “I have never registered it although I use a compatible device.”

^b^Worksite-A: Survey was conducted in November 2020. Data from 159 respondents were analyzed.

^c^Worksite-B: Survey was conducted in February 2021. Data from 188 respondents were analyzed.

^d^Coded as 2 = “female” and 1 = “male.”

^e^Coded as 5 = “60–69 years,” 4 = “50–59 years,” 3 = “40–49 years,” 2 = “30–39 years,” and 1 = “20–29 years.”

^f^Coded as 2 = “Married/partnered” and 1 = “Single/divorced/widowed.”

COCOA, COVID-19 Contact-Confirming Application.

## Discussion

This is the first attempt to examine the effectiveness of Ibaraki’s Amabie-chan in promoting infection prevention behavior and in mitigating fear of COVID-19. Although the surveys for each workplace were conducted at different periods, the findings were consistent between the two workplaces. We found a significant association between use of Ibaraki’s Amabie-chan and physical condition management. One of the plausible interpretations is that people who registered with it at shops or restaurants had simultaneously measured their body temperature by thermometers installed by the business owners. Another possible explanation is that people who visited shops and registered with Ibaraki’s Amabie-chan may have become worried about COVID-19 infection and felt an urgent need to measure their body temperature. It should also be noted that the frequency of visits to commercial facilities or restaurants, which was not examined in the current study, may have been acting as a potential covariate between them: those who stayed home did not have an opportunity to register. We found that Ibaraki’s Amabie-chan use was also associated with COCOA use. Since the two infection control systems have something in common, the relationship between them is convincing. In contrast to Ibaraki’s Amabie-chan, there were no significant correlation between COCOA use and physical condition management. One possible reason for the null correlation is that COCOA use is a passive behavior (just leaving once the setup has finished), whereas both Ibaraki’s Amabie-chan and body temperature measurement require relatively active behavior on the occasions.

Several limitations of the present study should be noted. Because of its cross-sectional design, we were unable to identify causal relationships. We used a convenience sample, not a representative sample. The valid response rate was low. Lastly, because Ibaraki’s Amabie-chan is a system unique to Ibaraki Prefecture, the generalizability of the current results is limited.

## Conclusions

Given the study’s limitations, our findings did not provide sufficient evidence for the effectiveness of Ibaraki’s Amabie-chan in regard to infection control behavior. Future study is needed to determine the overall effectiveness of the system more solidly. We also believe that future study is necessary to investigate the cost-effectiveness of running the local governments’ countermeasures against COVID-19, such as Ibaraki’s Amabie-chan. Such efforts will lead to a shift of human and financial resources to more effective countermeasures against COVID-19 nationwide.

## Abbreviations

COCOA: COVID-19 Contact-Confirming Application

## Declarations

### Availability of data and material

This study’s data are not publicly available. They are available from the corresponding author upon reasonable request and with the permission of the Occupational Health Committee, Tsukuba Science City Network.
